# Supplementary material for: Simulating high-speed solar wind streams from coronal holes using an L5-L1 configuration of Lagrangian points
Source: Sci Rep. 2025 Apr 15;15:12991. doi: 10.1038/s41598-025-97246-2 (PMC12000577; doi:10.1038/s41598-025-97246-2)
Supplement: Supplementary file 1 — Supplementary Information. [file 41598_2025_97246_MOESM1_ESM.pdf]

Simulating high-speed solar wind streams from coronal holes using an L5–L1 observational configuration

Tatiana Podladchikova[1,2,*], Astrid Veronig[2,3], Manuela Temmer[2], and Stefan J. Hofmeister[4]

[1]Skolkovo Institute of Science and Technology, Moscow, 121205, Russia
[2]Institute of Physics, University of Graz, Graz, 8010, Austria
[3]Kanzelhöhe Observatory for Solar and Environmental Research, University of Graz, Treffen, 9521, Austria
[4]Columbia University, New York, NY 10027, USA
*t.podladchikova@skol.tech

Supplementary Information. Figures for various spacecraft combinations and years

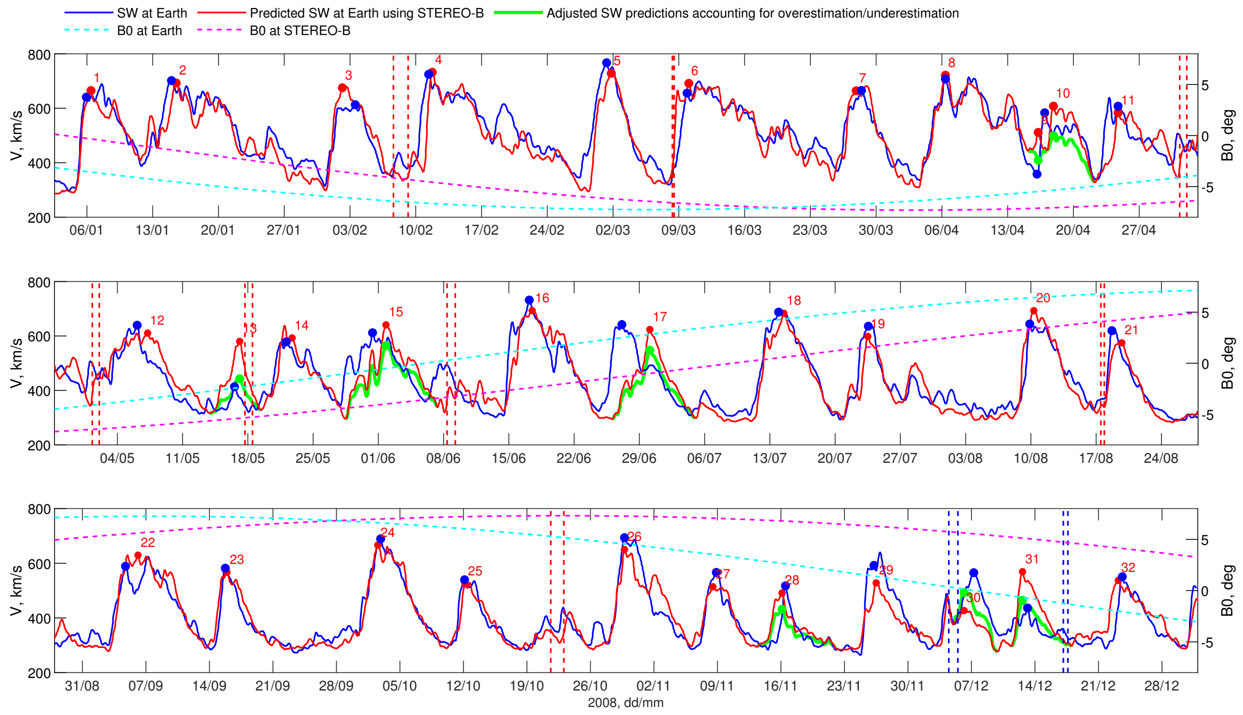

Supplementary Figure 1: The same as Figure 7 from the main text, but for predicting SW velocity at "L1" (Earth) using "L5" (STEREO-B) for 2008.

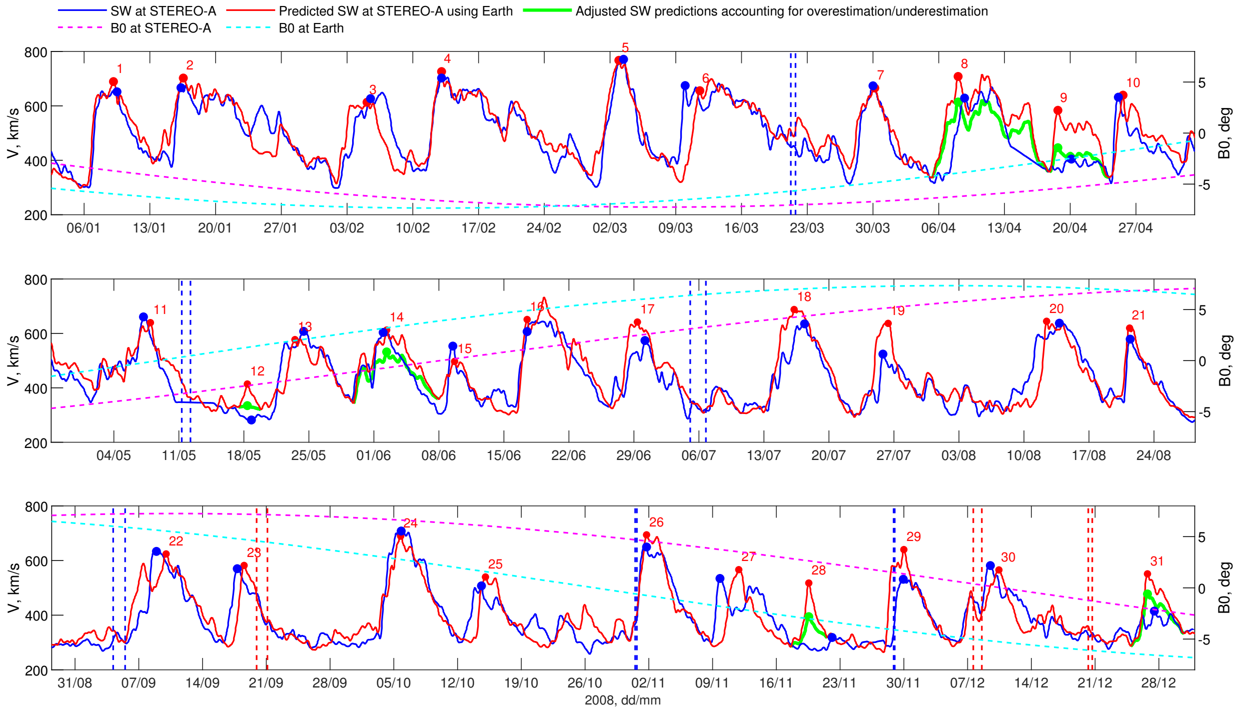

Supplementary Figure 2: The same as Figure 7 from the main text, but for predicting SW velocity at "L1" (STEREO-A) using "L5" (Earth) for 2008.

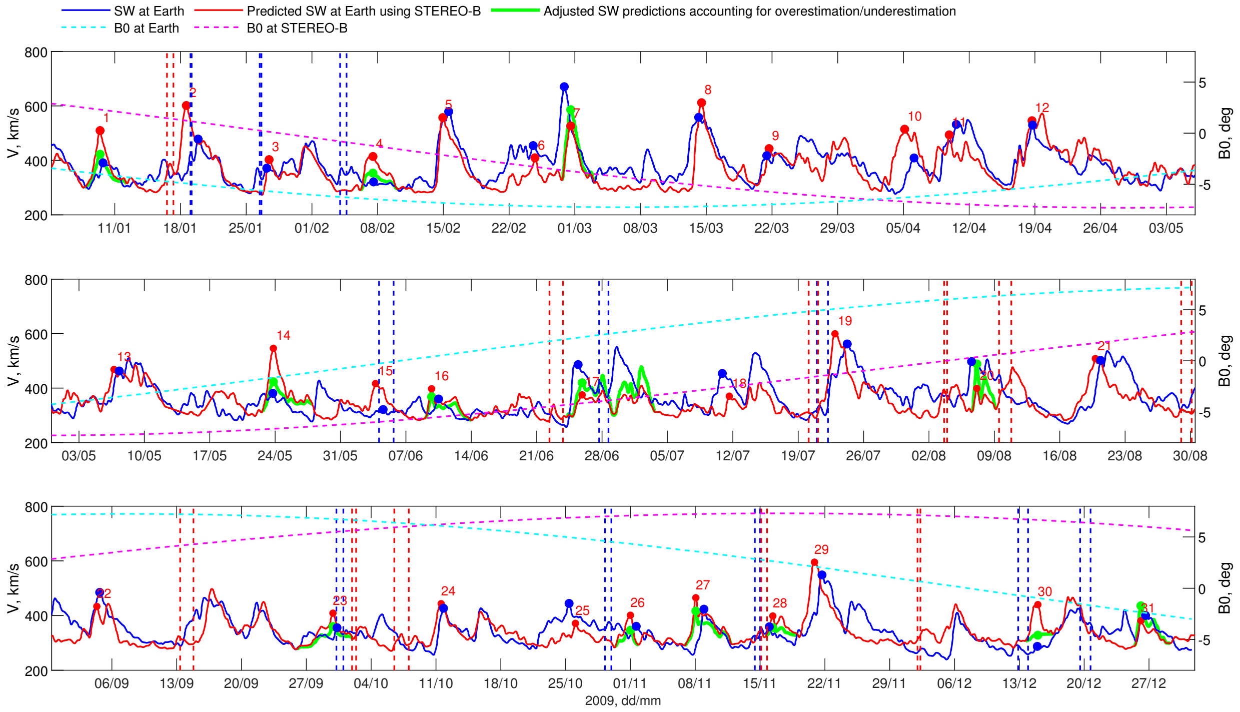

Supplementary Figure 3: The same as Figure 7 from the main text, but for predicting SW velocity at "L1" (Earth) using "L5" (STEREO-B) for 2009.

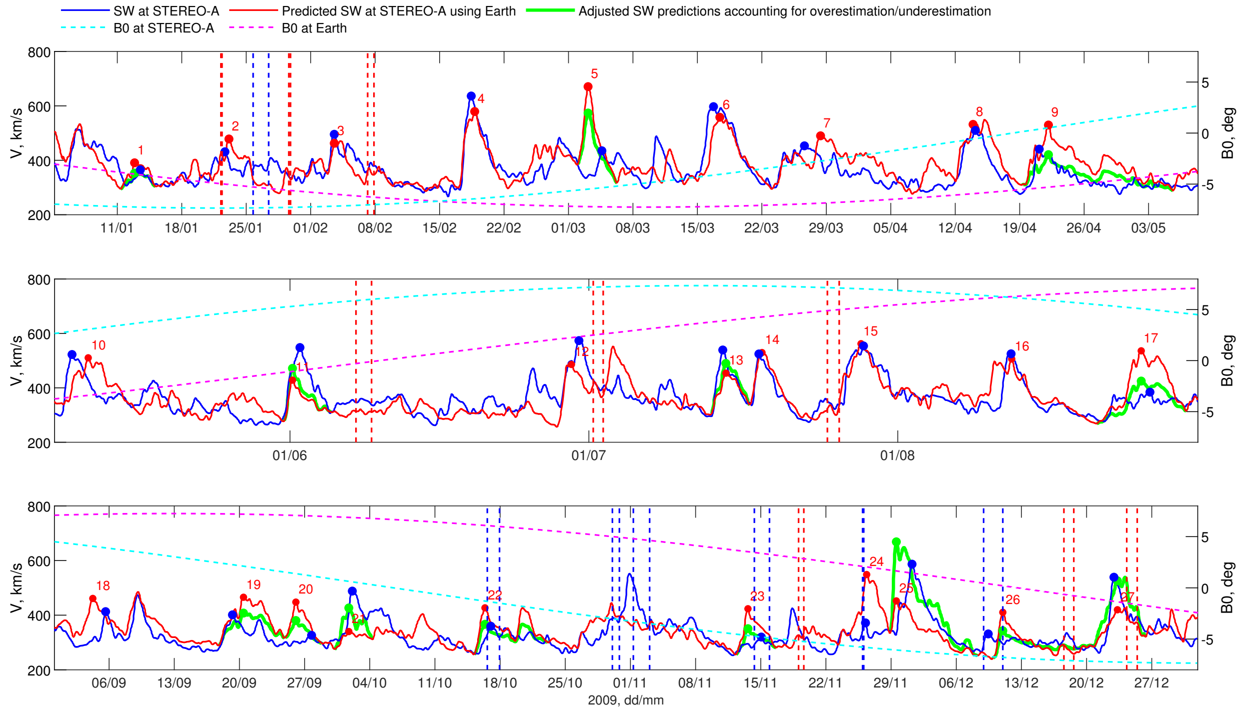

Supplementary Figure 4: The same as Figure 7 from the main text, but for predicting SW velocity at "L1" (STEREO-A) using "L5" (Earth) for 2009.

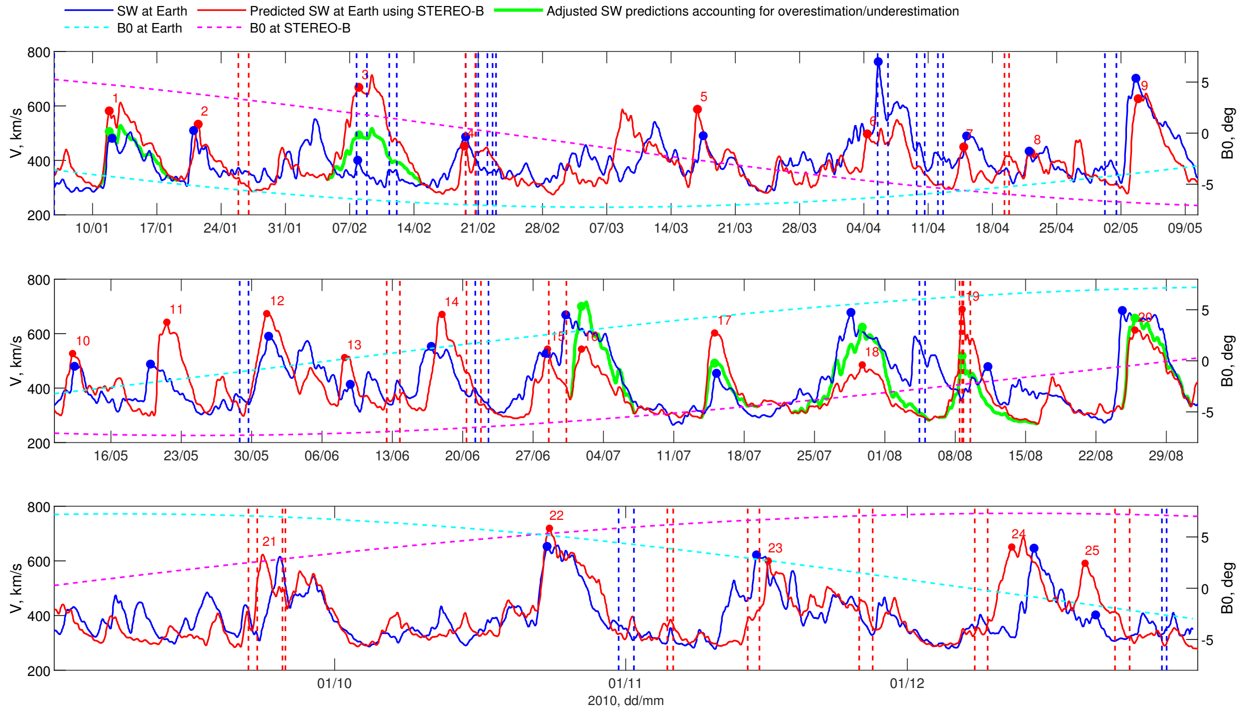

Supplementary Figure 5: The same as Figure 7 from the main text, but for predicting SW velocity at "L1" (Earth) using "L5" (STEREO-B) for 2010.

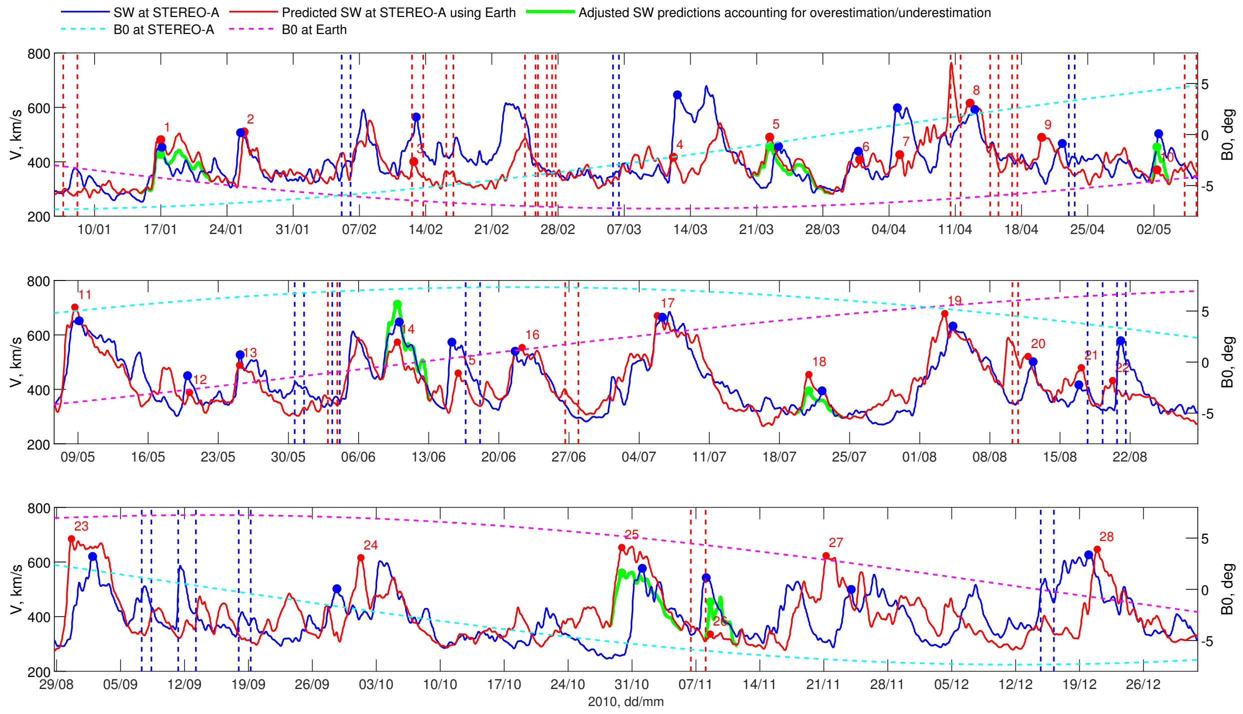

Supplementary Figure 6: The same as Figure 7 from the main text, but for predicting SW velocity at "L1" (STEREO-A) using "L5" (Earth) for 2010.
